# Salvianolic Acid A Attenuates Endoplasmic Reticulum Stress and Protects Against Cholestasis-Induced Liver Fibrosis via the SIRT1/HSF1 Pathway

**DOI:** 10.3389/fphar.2018.01277

**Published:** 2018-11-05

**Authors:** Jie Zhu, Ruiwen Wang, Ting Xu, Shuai Zhang, Yan Zhao, Zhenlu Li, Chao Wang, Junjun Zhou, Dongyan Gao, Yan Hu, Xiaofeng Tian, Jihong Yao

**Affiliations:** ^1^Department of Pharmacology, Dalian Medical University, Dalian, China; ^2^Department of General Surgery, Second Affiliated Hospital of Dalian Medical University, Dalian, China; ^3^Department of Gastroenterology, First Affiliated Hospital of Dalian Medical University, Dalian, China; ^4^Department of Pharmacy, Second Affiliated Hospital of Dalian Medical University, Dalian, China

**Keywords:** ER stress response, HSF1, liver fibrosis, salvianolic acid A, SIRT1

## Abstract

**Background:** Endoplasmic reticulum stress (ER stress) plays a critical role in the pathogenesis of liver fibrosis; thus, it can be a potential therapeutic target of fibrosis. However, the mechanism of ER stress regulation in fibrosis, particularly through sirtuin 1 (SIRT1), remains unclear. The objective of this study was to investigate the effect of SIRT1-mediated inhibition of ER stress in bile duct ligation (BDL)-induced liver fibrosis, and to explore the effect of salvianolic acid A (SalA) on BDL-induced liver fibrosis through SIRT1/heat shock factor 1 (HSF1) signaling.

**Materials and Methods:** We explored the effects of SalA on liver fibrosis and ER stress in BDL-induced liver fibrosis in rats and the human hepatic stellate cell line LX2 cells. The LX2 cells were treated with 20 ng of platelet-derived growth factor-BB homodimer (PDGF-BB) for 24 h, and then incubated in the absence or presence of SalA (25 μM) for 24 h.

**Results:** *In vivo*, SalA treatment alleviated BDL-induced liver injury and ER stress. Importantly, SalA treatment increased HSF1 expression and activity using a SIRT1-dependent mechanism. In LX2 cells, PDGF-BB induced ER stress and fibrosis were blocked by HSF1 overexpression. Furthermore, SIRT1 siRNA abrogated the SalA-mediated promotion of HSF1 deacetylation and expression, suggesting that SalA-mediated protection occurs by SIRT1 targeting HSF1 for deacetylation.

**Conclusion:** This is the first study to identify the SIRT1/HSF1 pathway as a key therapeutic target for controlling BDL-induced liver fibrosis and to show that SalA confers protection against BDL- and PDGF-BB-induced hepatic fibrosis and ER stress through SIRT1-mediated HSF1 deacetylation.

## Introduction

Liver fibrosis is a leading cause of liver health problems worldwide because of viral infection, alcoholism, chemical toxicity or metabolic and biliary disorders (Hernandez-Gea and Friedman, 2011; Sant’Anna et al., 2011). The BDL rat model has proven useful for research into curative antifibrotic effects (Lotersztajn et al., 2005). The mechanisms involved in the pathogenesis of fibrosis are not yet fully understood. Studies have suggested that endoplasmic reticulum (ER) and oxidative stress may play a critical role in the pathogenesis of liver cirrhosis (Wu et al., 2014). To cope with ER stress, mammalian cells have developed an adaptive mechanism called the UPR, signaled by three sensors residing on the ER membrane, IRE-1α, PERK, and ATF6 (Oakes and Papa, 2015). Over the past two decades, ER stress has emerged as an important mechanism involved in the pathogenesis of human diseases, such as diabetes, obesity, neurodegenerative disorders and cancer (Minamino and Kitakaze, 2010). More recently, ER stress has been observed in several liver diseases, including obesity-associated fatty liver disease, viral hepatitis and alcohol-induced liver injury (Malhi and Kaufman, 2011). The strengthening of the ER stress response is currently considered to contribute to the development of liver fibrosis (Koo et al., 2016). Therefore, understanding the molecular mechanisms that regulate ER stress may support the development of further therapeutic strategies for preventing and treating liver fibrosis.

Salvianolic acid A [3-(3′, 4′-dihydroxyphenyl)-2-hydroxypropanoic acid] is a naturally occurring plant polyphenolic acid extracted from *Salvia miltiorrhiza*. Studies have reported that SalA has a variety pharmacological effects, such as anti-oxidant, anti-fibrotic and anti-inflammatory activities (Liu et al., 2000; Tsai et al., 2010; Xu et al., 2014; Zhang et al., 2014). In our previous studies, we found that SalA diminishes concanavalin A (con A) -induced acute liver injury through a SIRT1/p66shc mechanism (Xu et al., 2013). Furthermore, our recent studies have shown that SalA ameliorates HFD-induced NAFLD by regulating the TXNIP/NLRP3 and TXNIP/CHREBP pathways (Ding et al., 2016). However, the influence of SalA on BDL-induced liver fibrosis is still unknown.

Sirtuin 1, a NAD-dependent class III histone deacetylase (HDAC) and the most interesting of the mammalian sirtuin family (SIRT1-7), affects physiological responses to aging-associated pathologies, including diabetes, liver steatosis, cardiovascular disease, neurodegeneration and various types of cancer (Lavu et al., 2009; Herranz and Serrano, 2010). Overexpression of SIRT1 or the SIRT1 activator resveratrol restores Aldo-induced MtD and EMT by upregulating PGC-1α in renal tubulointerstitial fibrosis (Yuan et al., 2012). However, recent studies have indicated that SIRT1 is a crucial regulator of fibroblast activation in systemic sclerosis (SSc), and TGF-β signaling and collagen expression can be effectively inhibited by the knockdown of SIRT1 (Zerr et al., 2016). Therefore, the role of SIRT1 in fibrosis is still under dispute. Studies have reported that Exendin-4, an agonist of the glucagon-like peptide 1 receptor, attenuates PA-induced ER stress via SIRT1 in HepG2 cells (Lee et al., 2014), and SIRT1 attenuates hepatic steatosis, ameliorates insulin resistance and restores glucose homeostasis through the inhibition of mTORC1 and ER stress in human type 2 diabetes (Li et al., 2011). Thus, we hypothesized that SIRT1 may suppress ER stress in BDL-induced liver fibrosis, thereby affording a novel therapeutic target.

Heat shock factor 1 plays an important role in protecting cells from protein-damaging stress associated with misfolded proteins by rapidly translocating to hsp genes to induce their expression, counteracting cell stress and regulating disease states and aging (Xu et al., 2005; Westerheide et al., 2009). Studies have reported that SIRT1-mediated HSF1 deacetylation can prolong HSF1 binding to the promoter of the heat shock gene Hsp70, positively regulate DNA binding activity and enhance the HSR (Westerheide et al., 2009). Moreover, it has been reported that HSR can relieve stress in UPR-deficient cells by affecting multiple ER activities (Liu and Chang, 2008) and that HSF1 knockdown enhances heat-induced ER stress and subsequent cell death in human glioma A172 cells (Liu et al., 2012). Thus, we hypothesized that SIRT1 may relieve ER stress through HSF1 deacetylation. Decreased levels of vitamin B12 strongly induce ER stress in N1E115 dopaminergic cells by the greater acetylation of HSF1 through decreased SIRT1 expression (Ghemrawi et al., 2013). Angiotensin II (ANG II) activates JNK to degrade SIRT1, resulting in HSF1 acetylation, which alleviates the repression of IGF-IIR gene expression by HSF1 and protects cardiomyocytes (Huang et al., 2014). The SIRT1 and SIRT2 inhibitors EX527 and AGK2, respectively, suppress HeLa cell proliferation by inhibiting the expression of HSF1 and HSP27 and inducing HSF1 ubiquitination (Kim et al., 2016). However, the role of SIRT1/HSF1 in fibrosis is unknown.

The purposes of this study were as follows: (1) to test whether the signaling mechanisms underlying the ER stress inhibition effects are associated with the SIRT1/HSF1 pathway in BDL-induced liver fibrosis; (2) to study whether SalA inhibits ER stress by up-regulating SIRT1; and (3) to explore the role of the ER stress inhibition effect of SalA in protecting against BDL-induced liver fibrosis.

## Materials and Methods

### Experimental Animals and Reagents

Male Sprague-Dawley rats ranging from 180 to 220 g were obtained from the Experimental Animal Center of Dalian Medical University (Dalian, China). SalA (98% purity) was purchased from Shanghai Winherb Medical Science Co., Ltd. (Shanghai, China) and dissolved in water. Briefly, rats were anesthetized with a pentobarbital intraperitoneal injection (50 mg/kg body weight). After midline abdominal incision, common bile duct (CBD) was doubly ligated with 4-0 silk sutures and cut with a #11 blade. Sham operation was just exposure of the CBD by bypassing the cotton applicator below the foramen of Winslow. After saline irrigation, abdominal closure was performed using a continuous 3-0 silk. After the procedure, the animals were divided into five groups: (1) control, (2) control + SalA (20 mg/kg/day), (3) BDL, (4) BDL + SalA (10 mg/kg/day) and (5) BDL + SalA (20 mg/kg/day). 24 h after surgery, the rats in groups 2, 4, and 5 were administrated SalA for 3 weeks, whereas those of groups 1 and 3 were intragastrically given the same amount of saline. At the end of the experiment, blood and liver samples were harvested for analysis. All the procedures were performed in compliance with the Institute’s guidelines and the Guide for the Care and Use of Laboratory Animals. The study was approved by the institutional animal care committee of Dalian Medical University.

### Biochemical Assays

Serum samples were separated by centrifugation at 2,300 ×*g* for 10 min and were kept at -20°C until analysis. The serum levels of ALT, AST, and total bilirubin (Tbil) were determined using commercial kits from the Nanjing Jiancheng Bioengineering Institute (Nanjing, China). All of the procedures were carried out according to the manufacturers’ instructions.

### Liver Histological Examination

Paraffin-embedded liver tissue samples were cut into 5-μm thick sections for hematoxylin and eosin staining, and the sections were then examined by light microscopy. This procedure stains nuclei black, cytoplasm and muscle fibers red and ECM components blue. The degree of fibrosis was measured using Masson’s trichrome staining and then examined by light microscopy. Fibrotic rate represent the ratio of the Masson’s positive area to the total examined area (Gracia-Sancho et al., 2011; Chen et al., 2015).

### Cell Culture and Treatment

The human hepatic stellate cell line LX2 was cultured in modified Eagle’s medium (MEM) supplemented with 10% (v/v) fetal bovine serum (FBS) (Gibco, Carlsbad, CA, United States). The cells were kept at 37°C in a humidified incubator with 5% CO_2_. PDGF-BB (Sigma no. P4056) was purchased from Sigma-Aldrich (St. Louis, MO, United States). Tunicamycin was purchased from Dalian Meilun Biological Technology Co., Ltd. (Dalian, China) and salubrinal was purchased from Selleck (Houston, TX, United States). The *in vitro* model of fibrosis was established by treating LX2 cells with PDGF-BB, and then LX2 cells were incubated in the absence or presence of SalA (25 μM) for 24 h, which were determined by preliminary experiment and reported (Lin et al., 2006; Ding et al., 2016). *In vitro*, LX2 cells were stimulated with 20 ng of PDGF-BB for 24 h. After incubation with 10 μM of EX527 for 6 h, the LX2 cells were subjected to 20 ng of PDGF-BB and/or 25 μM of SalA stimulation as needed.

### Western Blot Analysis

Equal amounts of protein samples were separated by 10–15% SDS-PAGE and transferred to PVDF membranes (Millipore, Bedford, MA, United States). After blocking, the membranes were immunoblotted with primary antibodies that were specific for SIRT1, acetyl-lysine (Abcam Ltd., Cambridge, United Kingdom), ATF6, GRP78, COL1A2, and α-SMA (Proteintech Group, Wuhan, China), PERK and ATF4 (Wanlei Biotech, Shenyang, China), HSF1 (Cell Signaling Technology, Saint-Quentin-en-Yvelines, France), P-PERK, P-IRE1-α and IRE1-α (Bioss, Beijing, China), β-actin (Santa Cruz Biotechnology, Santa Cruz, CA, United States) and HSP27 (ABclonal, Wuhan, China). After washing, the membranes were incubated with the appropriate secondary antibodies. The membranes were then exposed to enhanced chemiluminescence-plus reagents (Beyotime Institute of Biotechnology, Hangzhou, China). The emitted light was captured by a BioSpectrum 410 multispectral imaging system with a Chemi 410 HR camera and analyzed with Gel-Pro Analyzer Version 4.0 (Media Cybernetics, Rockville, MD, United States).

### Analysis of Acetylated HSF1 by Immunoprecipitation

A sufficient amount of anti-HSF1 antibody (Cell Signaling Technology, Saint-Quentin-en-Yvelines, France) was added to 200 μg of protein and gently rotated at 4°C overnight. Next, the immunocomplex was captured by adding 25 μL of protein A + G agarose beads (Beyotime, Shanghai, China) and gently rotating the mixture at 4°C for 3 h. The mixture was then centrifuged at 1,500 ×*g* for 5 min at 4°C. The precipitate was washed three times with ice-cold phosphate-buffered saline, resuspended in 1 × sample buffer and boiled for 5 min to dissociate the immunocomplex from the beads. Finally, the supernatant was collected by centrifugation and subjected to Western blotting.

### siRNA and Plasmid Transfection

LX2 cells were seeded on 6-well plates at a density of 1 × 10^5^ cells/dish. When the confluence reached 50–60%, the LX2 cells were transfected with a specific SIRT1 siRNA, a specific HSF1 siRNA (100 nM), HSF1 plasmid or with non-binding control siRNA (100 nM) using Lipofectamine 3000 (Invitrogen, Karlsruhe, Germany) according to the manufacturer’s instructions. The SIRT1 siRNA sequences were sense 5′-CCCUGUAAAGCUUUCAGAAdtdt-3′ and antisense 5′-UUCUGAAAGCUUUACAGGGdtdt-3′ (Genepharma, Shanghai, China). The HSF1 siRNA sequences were sense 5′-GCGGCAGCUCAACAUGUAUdTdT-3′ and antisense 5′-AUACAUGUUGAGCUGCCGCdTdT-3′ (Genepharma, Shanghai, China).

### Statistical Analysis

The results are expressed as the mean ± SD. Statistical analyses were performed using GraphPad Prism (version 5.0; GraphPad Prism Software, La Jolla, CA, United States). The data were analyzed with a two-tailed unpaired Student’s *t*-test or one-way analysis of variance to determine the statistical significance of differences between the groups. *P*-values < 0.05 were considered to indicate statistical significance.

## Results

### SalA Diminishes BDL-Induced Liver Injury and Liver Fibrosis

**FIGURE 1:**
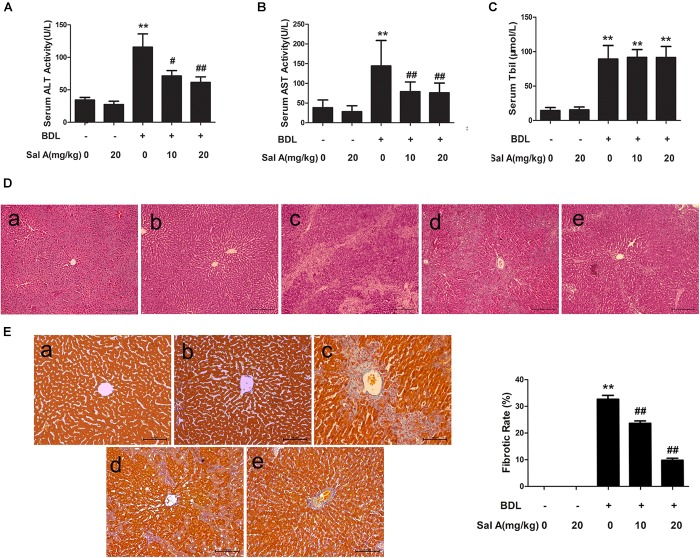
SalA diminishes BDL-induced liver injury and hepatic fibrosis. **(A,B)** Serum levels of alanine aminotransferase and aspartate aminotransferase (AST). **(C)** Serum Tbil. The results are presented as the mean ± SD (*n* = 8). ^∗∗^*P* < 0.01 vs. the control group, ^#^*P* < 0.05 vs. the BDL group, ^##^*P* < 0.01 vs. the BDL group. **(D,E)** HE staining and Masson’s trichrome staining of liver sections from the experimental groups: a, control; b, control + SalA (20 mg/kg); c, BDL; d, BDL + SalA (10 mg/kg) and e, BDL + SalA (20 mg/kg). HE-stained sections were photographed at 100× magnification, and Masson-stained sections were photographed at 200× magnification. Fibrotic rate represents the ratio of the Masson’s positive area to the total examined area. The results are presented as the mean ± SD (*n* = 8). ^∗∗^*P* < 0.01 vs. the control group, ^##^*P* < 0.01 vs. the BDL group.

As shown in Figures 1A–C, BDL caused a significant increase in the levels of serum ALT, AST, and Tbil compared with sham-operated rats. The ligation of the common bile duct significantly increased systemic bilirubin levels by more than threefold, suggesting that clear-cut cholestasis was induced in this model. Bilirubin levels in mice treated with SalA were not different from those in vehicle-treated animals after BDL, indicating that the degree of cholestasis was similar in all experimental groups (Figure 1C). SalA treatment remarkably inhibited ALT and AST activities induced by BDL in a dose-dependent manner, but did not affect ALT and AST levels in control rats, suggesting a protective effect for SalA against BDL-induced liver injury. In agreement, according to HE staining (Figure 1D), there was marked architectural distortion of the lobular structure and extensive bile duct proliferation infiltrating into hepatocytes with periportal fibrogenesis and occasional fibrogenesis in the parenchymal area, suggesting the activation of HSCs. SalA treatment reduced these pathological changes compared with the BDL group. Because liver fibrosis is characterized by an increased accumulation of ECM, mainly collagens, we assessed the collagen contents based on Masson’s trichrome staining and Western blotting for type I collagen. Consistent with the histopathology, SalA markedly suppressed collagen accumulation and reduced fibrosis in BDL rat livers (Figure 1E). This suggests that SalA improves the histological changes and reduces ECM accumulation in liver fibrosis.

### SalA Alleviates Liver Fibrosis by Suppressing ER Stress

**FIGURE 2:**
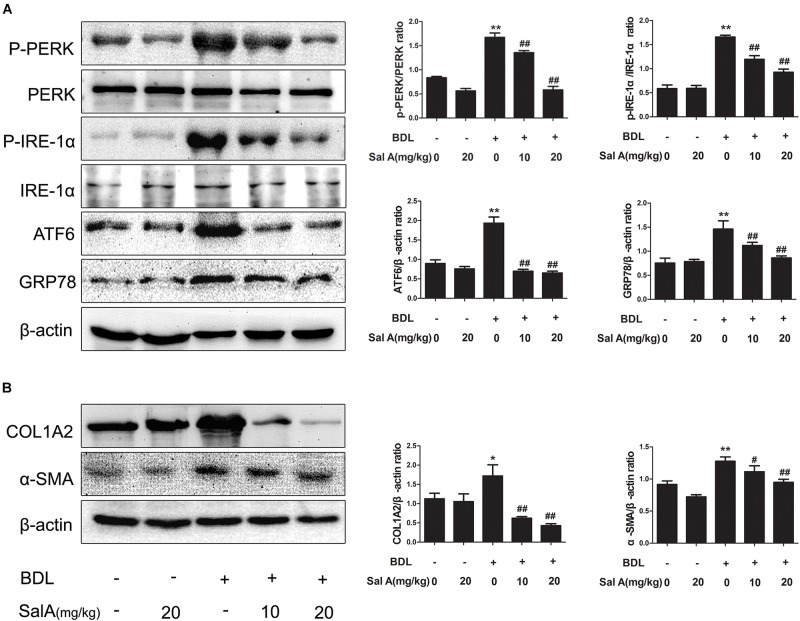
SalA suppresses BDL-induced liver fibrosis and the endoplasmic reticulum (ER) stress response *in vivo*. **(A,B)** Western blot analysis of phosphorylated PERK (P-PERK), PERK, phosphorylated IRE-1 α (P-IRE-1 α), IRE-1 α, transcription factor 6 (ATF6), GRP78, COL1A2 and α-SMA in the rat livers. The results are presented as the mean ± SD (*n* = 3). ^∗^*P* < 0.05 vs. the control group, ^∗∗^*P* < 0.01 vs. the control group, ^#^*P* < 0.05 vs. the BDL group, ^##^*P* < 0.01 vs. the BDL group.

Hepatic stellate cells (HSCs) activation contribute to the development of liver fibrosis (Hernandez-Gea and Friedman, 2011), and ER stress induces fibrogenic genes and promotes liver fibrosis in HSCs (Malhi and Kaufman, 2011; Zhao et al., 2018). Thus, we next measured whether SalA can suppress ER stress and alleviate liver fibrosis. We measured the protein levels of three ER stress transducers, P-PERK, P-IRE-1α, and ATF6, to explore the influence of BDL on the UPR. To further evaluate the consequences of ER stress produced by BDL, we measured the levels of GRP78, the central mediator of ER stress (Figure 2A). Indeed, we found that the protein levels of P-PERK/PERK, P-IRE-1α/IRE-1α, ATF6 and GRP78 were remarkably increased in the BDL group, whereas SalA treatment prevented the increase in levels of ER stress proteins. We also measured the levels of the fibrosis markers COL1A2 and α-SMA, and the results were the same (Figure 2B).

**FIGURE 3:**
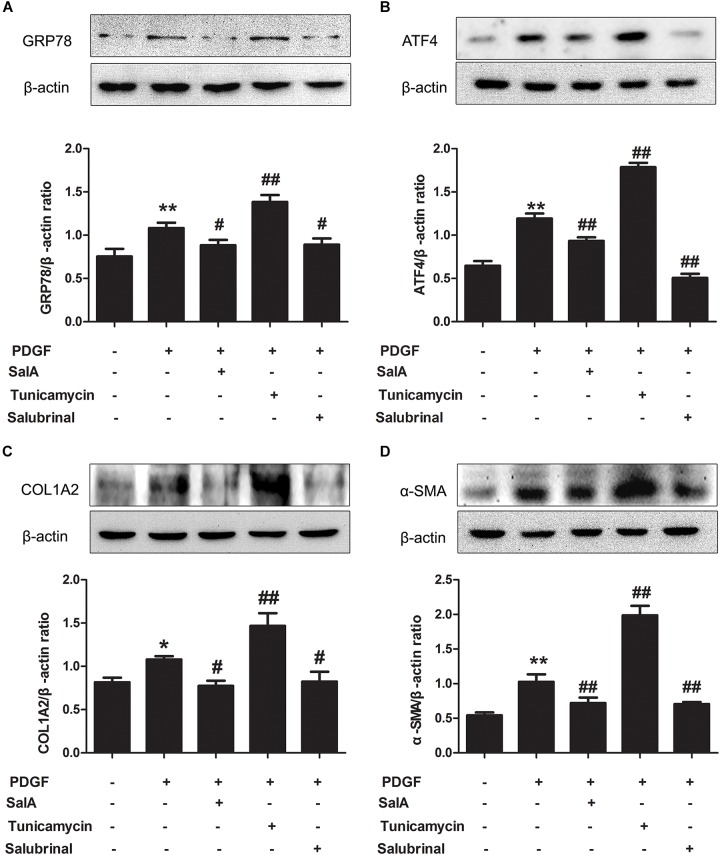
SalA alleviates liver fibrosis by suppressing ER stress *in vitro*. **(A–D)** LX2 cells were exposed to 20 ng/ml of PDGF-BB with 25 μM of SalA or 2.5 μg/ml of Tunicamycin or 30 μM of salubrinal for 24 h. The protein levels of GRP78, ATF4, COL1A2 and α-SMA were evaluated by Western blotting. The results are presented asthe mean ± SD (*n* = 3). ^∗^*P* < 0.05 vs. the control group, ^∗∗^*P* < 0.01 vs. the control group, ^#^*P* < 0.05 vs. the PDGF-BB group, ^##^*P* < 0.01 vs. the PDGF-BB group.

To further strengthen our hypothesis, an ER stress inducer, tunicamycin, was used to directly trigger ER stress in LX2 cells. We found that tunicamycin could stimulate ER stress, which activated HSCs and thereby promoted liver fibrosis. However, salubrinal, an ER stress inhibitor, reversed these trends. As expected, SalA had the same effect as salubrinal (Figures 3A–D). Taken together, SalA alleviates liver fibrosis by suppressing ER stress.

### SalA Mediates Protection Against BDL- and PDGF-BB-Induced Hepatic Fibrosis and ER Stress by Up-Regulating SIRT1

**FIGURE 4:**
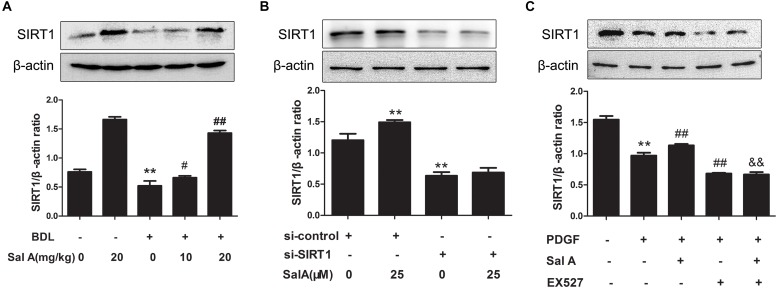
Effects of SalA on SIRT1 protein expression *in vivo* and *in vitro*. **(A)** Western blot analysis of the hepatic SIRT1 protein level in rats. The results are presented as the mean ± SD (*n* = 3). ^∗∗^*P* < 0.01 vs. the control group, ^#^*P* < 0.05 vs. the BDL group, ^##^*P* < 0.01 vs. the BDL group. **(B)** LX2 cells were transfected with control siRNA or SIRT1 siRNA, and the transfected cells were exposed to 25 μM of SalA for 24 h. The SIRT1 protein level was then measured by Western blotting. The results are presented as the mean ± SD (*n* = 3). ^∗∗^*P* < 0.01 vs. the control group. **(C)** LX2 cells were pretreated with 10 μM of EX527 for 6 h before being exposed to 25 μM of SalA and/or 20 ng/ml of PDGF-BB. The protein level of SIRT1 was evaluated by Western blotting. The results are presented as the mean ± SD (*n* = 3). ^∗∗^*P* < 0.01 vs. the control group, ^##^*P* < 0.05 vs. the PDGF-BB group, ^&&^*P* < 0.01 vs. the PDGF-BB group treated with 25 μM of SalA.

Sirtuin 1, a transcriptional silencing factor at the silent mating loci, is known to play a role in fibrosis and ER stress (Chang et al., 2016). SalA treatment effectively attenuated hepatic ER stress in BDL rats. To further explore the mechanism, we tested whether the effects exerted by SalA involved SIRT1. We found that hepatic SIRT1 protein level was remarkably decreased in BDL rats, and SalA reversed this loss of SIRT1 (Figure 4A). In parallel with the above in hepatic tissue, we used PDGF-BB stimulated LX2 cells with SIRT1 reduction, and SalA treatment caused SIRT1 up-regulation in this cell model. Importantly, SalA-mediated SIRT1 up-regulation was mostly abrogated upon SIRT1 small interfering RNA (siRNA) transfection in LX2 cells (Figure 4B).

**FIGURE 5:**
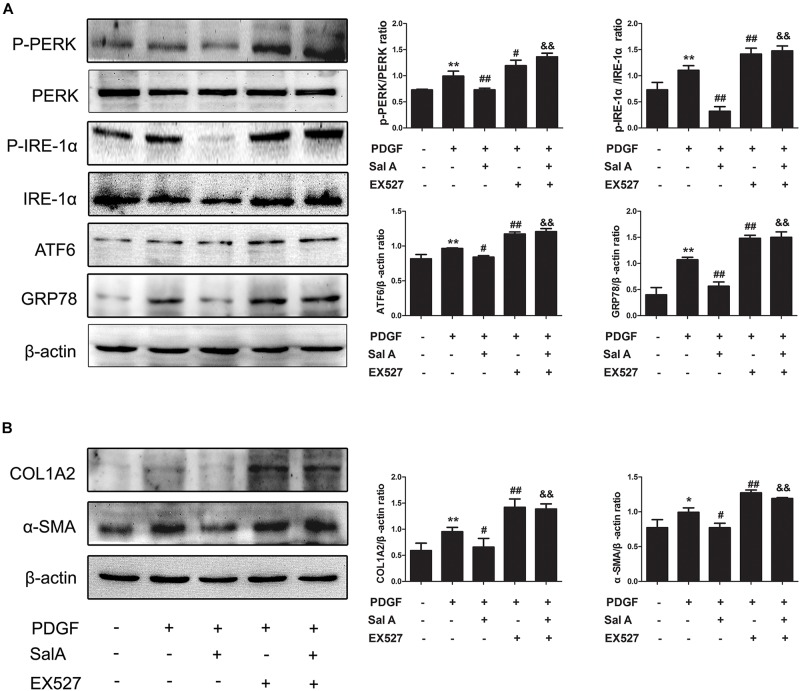
SalA mediates protection against BDL- and PDGF-BB-induced hepatic fibrosis and ER stress through SIRT1 up-regulation. LX2 cells were pretreated with 10 μM of EX527 for6 h before being exposed to 25 μM of SalA and/or 20 ng/ml of PDGF-BB. **(A,B)** Western blot analysis of the levels of phosphorylated PERK (P-PERK), PERK, phosphorylated IRE-1 α (P-IRE-1 α), IRE-1 α, transcription factor 6 (ATF6), GRP78, COL1A2 and α-SMA. The results are presented as the mean ± SD (*n* = 3). ^∗^*P* < 0.05 vs. the control group, ^∗∗^*P* < 0.01 vs. the control group,^#^*P* < 0.05 vs. the PDGF-BB group, ^##^*P* < 0.01 vs. the PDGF-BB group, ^&&^*P* < 0.01 vs. the PDGF-BB group treated with 25 μM of SalA.

To further evaluate whether SIRT1 contributed to SalA-mediated protection *in vitro*, LX2 cells were pretreated with the specific SIRT1 antagonist EX527 before being exposed to SalA and/or PDGF-BB. The PDGF-BB treatment remarkably decreased SIRT1 expression, while the SalA treatment significantly increased SIRT1 protein levels (Figure 4C). Moreover, the protein levels of P-PERK/PERK, P-IRE-1α/IRE-1α, ATF6 and GRP78 were remarkably increased by PDGF-BB treatment, which were inhibited by SalA treatment. PDGF-BB treatment also increased the protein levels of COL1A2 and α-SMA, and SalA decreased the levels of the above fibrosis markers. Whereas the SalA-mediated protection was abrogated upon EX527 treatment in LX2 cells (Figures 5A,B). Thus, these results suggest that SalA-induced protection against BDL- and PDGF-BB-induced hepatic fibrosis and ER stress is mediated by the up-regulation of SIRT1.

### SalA-Mediated Protection Against Liver Fibrosis and ER Stress Is Related to HSF1 Up-Regulation

**FIGURE 6:**
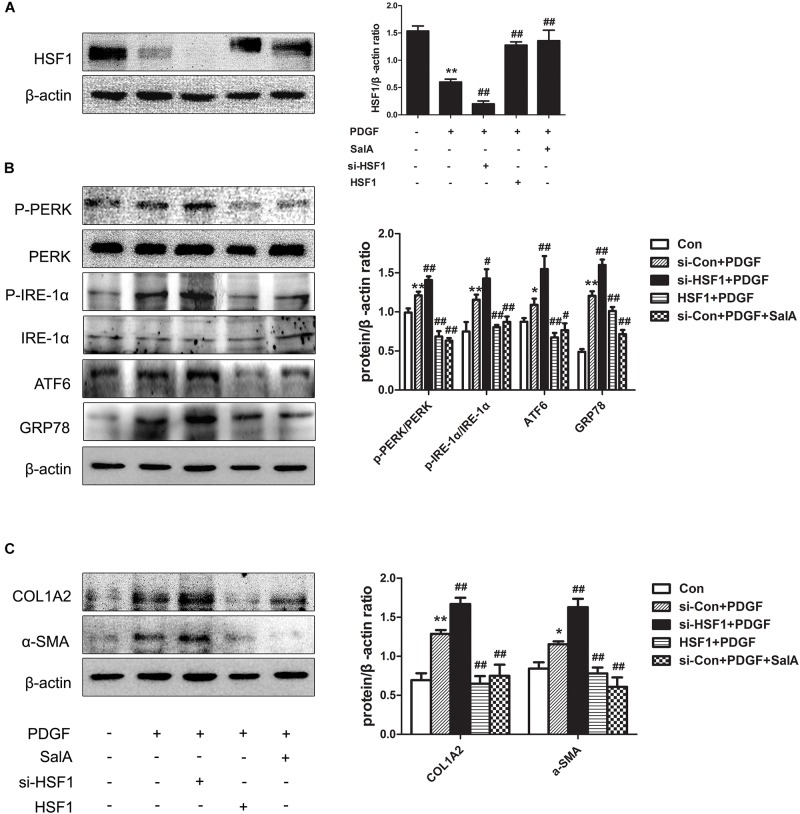
SalA-mediated protection is related to HSF1 up-regulation. LX2 cells were transfected with HSF1 siRNA or HSF1, and the transfectedcells were exposed to 20 ng/ml of PDGF-BB for 24 h. **(A)** The HSF1 protein level was then measured by Western blotting. The results are presented as the mean ± SD (*n* = 3). ^∗∗^*P* < 0.01 vs. the control group, ^##^*P* < 0.01 vs. the PDGF-BB group. **(B,C)** Western blot analysis of the protein levels of phosphorylated PERK (P-PERK), PERK, phosphorylated IRE-1 α (P-IRE-1 α), IRE-1 α, transcription factor 6 (ATF6), GRP78, COL1A2 and α-SMA. The results are presented asthe mean ± SD (*n* = 3). ^∗^*P* < 0.05 vs. the control group, ^∗∗^*P* < 0.01 vs. the control group, ^#^*P* < 0.05 vs. the PDGF-BB group, ^##^*P* < 0.01 vs. the PDGF-BB group.

HSF1 is the master regulator of the HSR and alters the transcription of a large number of genes, it also acts as a regulator of disease states and aging (Xu et al., 2005). To ascertain the role of HSF1 in BDL-induced fibrosis in the current study, the overexpression of HSF1 and immunoprecipitation were used to confirm our hypothesis. We found that after treatment with PDGF-BB, the expression of HSF1 was decreased, and ER stress and fibrosis were induced, while these changes were blocked by HSF1 overexpression and SalA treatment in LX2 cells (Figures 6A–C). These results demonstrate that HSF1 plays a critical role in the development of PDGF-BB-induced fibrosis and ER stress, and SalA-mediated protection is related to HSF1 up-regulation.

### SalA-Mediated Protection Against Liver Fibrosis and ER Stress Depends on SIRT1 Targeting HSF1 for Deacetylation

**FIGURE 7:**
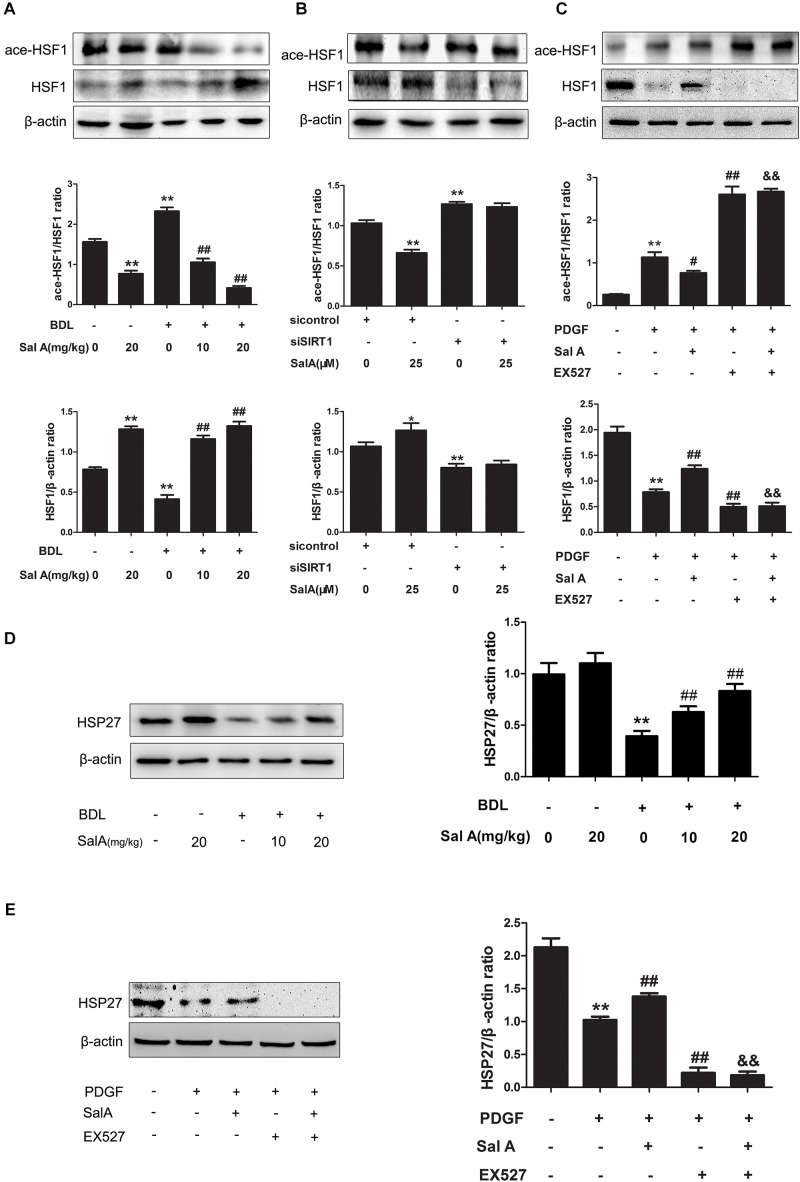
SalA-mediated protection depends on SIRT1 targeting HSF1 for deacetylation. **(A)** Western blot analysis of the protein levels of HSF1and ace-HSF1 in the rat livers. The results are presented as the mean ± SD (*n* = 3). ^∗∗^*P* < 0.01 vs. the control group, ^##^*P* < 0.01 vs. the BDL group. **(B)** LX2 cells were transfected with control siRNA or SIRT1 siRNA, and the transfected cells were exposed to 25 μM of SalA for 24 h. HSF1and ace-HSF1 levels were then measured by Western blotting. The results are presented as the mean ± SD (*n* = 3). ^∗^*P* < 0.05 vs. the control group, ^∗∗^*P* < 0.01 vs. the control group. **(C)** LX2 cells were pretreated with 10 μM of EX527 for 6 h before being exposed to 25 μM of SalA and/or 20 ng/ml of PDGF-BB. Western blot analysis of the protein levels of HSF1and ace-HSF1. The results are presented as the mean ± SD (*n* = 3). ^∗∗^*P* < 0.01 vs. the control group, ^#^*P* < 0.05 vs. the PDGF-BB group, ^##^*P* < 0.01 vs. the PDGF-BB group, ^&&^*P* < 0.01 vs. the PDGF-BB group treated with 25 μM of SalA. **(D)** Western blot analysis of the protein level of HSP27 in the rat livers. The results are presented as the mean ± SD (*n* = 3). ^∗∗^*P* < 0.01 vs. the control group, ^##^*P* < 0.01 vs. the BDL group. **(E)** LX2 cells were pretreated with 10 μM of EX527 for 6 h before being exposed to 25 μM of SalA and/or 20 ng/ml of PDGF-BB. Western blot analysis of the protein level of Hsp27. The results are presented as the mean ± SD (*n* = 3). ^∗∗^*P* < 0.01 vs. the control group, ^##^*P* < 0.01 vs. the PDGF-BB group, ^&&^*P* < 0.01 vs. the PDGF-BB group treated with 25 μM of SalA.

Further regulation of HSF1 is provided by posttranslational modifications, including phosphorylation, acetylation, sumoylation and oxidation of cysteines to disulfide bridges (Hentze et al., 2016). We thus examined whether the process of HSF1 translocation and release is regulated by SIRT1-mediated deacetylation. As shown in Figure 7A, the BDL group had increased acetylation of HSF1 compared to the control group, while SalA reduced the levels of acetylated HSF1 in a dose-dependent manner. In particular, to assess whether SalA-induced protection is mediated by SIRT1 through targeting HSF1 for deacetylation, we examined the effect of SalA on the status of HSF1 acetylation following SIRT1 siRNA treatment. In contrast to the control siRNA treatment, SIRT1 knockdown markedly reduced the release of HSF1 and deacetylated HSF1. However, SalA counteracted the decrease of HSF1 and significantly reduced the proportion of acetylated HSF1, and the SalA-mediated down-regulation of acetylated HSF1 was blocked by SIRT1 siRNA (Figure 7B). Furthermore, PDGF-BB exposure remarkably increased the level of acetylated HSF1. However, acetylated HSF1 was significantly decreased by SalA treatment, but this effect was blocked by EX527 (Figure 7C). Importantly, the deacetylation of HSF1 was shown to enhance the HSF1 DNA-binding activity and lead to an abundant HSR. SalA markedly increased the expression of heat shock protein HSP27 in BDL rat livers (Figure 7D). As expected, SalA also increased the level of the heat shock protein HSP27 in LX2 cells, but this effect was blocked by EX527 (Figure 7E). These data demonstrate that the SalA-mediated increase of HSF1 deacetylation and activity are partly achieved through the up-regulation of SIRT1.

## Discussion

Fibrosis is a chronic liver damage process that aim to maintain organ integrity upon extensive necrosis or apoptosis occurs. The regression of fibrosis and cirrhosis has been described in relation to chronic HBV or HCV infection, alcoholic steatohepatitis, NASH, chemical toxicity and biliary diseases (Friedman, 2008; Sant’Anna et al., 2011). Significant improvements in the treatment of chronic liver disease have accelerated interest in exploiting the mechanisms underlying hepatic fibrosis and its resolution. The pathogenic process of fibrosis is strongly linked to the accumulation of extracellular matrix (ECM), inflammation, stellate cell activation and ER stress (Friedman, 2008; Hernandez-Gea and Friedman, 2011; Wu et al., 2014). However, effective drugs to prevent or revert hepatic fibrosis are still lacking (Fagone et al., 2016). SalA, one of the water soluble components extracted from *Salvia miltiorrhiza*, has been reported to have the potent activity against fibrosis on account of its anti-oxidative, pro-apoptotic and, anti-proliferative properties and its ability to inhibit collagen synthesis (Liu et al., 2000; Lin et al., 2006; Tsai et al., 2010; Pan et al., 2014). In this study, we discovered that SalA can diminish BDL- and PDGF-BB-induced liver injury and liver fibrosis by suppressing ER stress. Moreover, we determined that the SIRT1/HSF1 pathway is involved in the protective effects of SalA against BDL- induced liver fibrosis.

SIRT1, a NAD-dependent deacetylase, plays a central role in the pathogenesis of fibrosis. SIRT1 contributes to apoptosis and suppresses the proliferation, migration and activation of HSCs (Qi et al., 2015; Wu et al., 2015). It also represses the binding of SMAD3 to fibrogenic gene promoters (Sun et al., 2016). Several pharmacological SIRT1 activators or SIRT1 inhibitors have been found to improve or contribute fibrosis (Sun et al., 2016; Xiao et al., 2016). Importantly, we have previously shown that SalA is a potent activator of SIRT1 (Xu et al., 2013). In this study, we found that SIRT1 expression was significantly decreased during BDL-induced liver fibrosis. Furthermore, SalA increased SIRT1 expression, suggesting that SalA may protect against BDL-induced liver fibrosis by up-regulating SIRT1. To further evaluate such a possibility, we used SIRT1 siRNA to stimulate LX2 cells. As expected, SIRT1 siRNA treatment prevented the up-regulation of SIRT1 by SalA. Accordingly, LX2 cells were pretreated with EX527 before being exposed to PDGF-BB, and the SalA-mediated protection was abrogated upon EX527 treatment *in vitro*. Therefore, SIRT1 up-regulation is related to the therapeutic action of SalA against BDL- and PDGF-BB-induced fibrosis.

Recently, ER stress has emerged as an important factor in the development of organ fibrosis through the activation of TGF-β-induced myofibroblasts, pro-apoptotic pathways, epithelial-mesenchymal transition and inflammatory responses (Lenna and Trojanowska, 2012; Heindryckx et al., 2016). Furthermore, the chemical chaperon 4-phenylbutyrate, an inhibitor of ER stress, acts as an ER chaperone to ameliorate ER stress-induced renal tubular cell apoptosis and renal fibrosis (Liu et al., 2016). As ER stress is increasingly identified as a mediator of inflammation and fibrotic remodeling, blocking ER stress may be a promising therapeutic approach to treating fibrosis. We further explored the anti-ER stress pathway, which may be related to SIRT1. HSF1 has been reported to be deacetylated by SIRT1, which regulates DNA binding activity and enhances HSR (Westerheide et al., 2009). As reported that, HSR is a cellular stress response that senses protein misfolding and restores protein folding homeostasis, or proteostasis, which can relieve ER stress through multiple pathways (Ma et al., 2017). HSF1, as the master regulator of the HSR, protects cells and organisms against severe stress insults (Xu et al., 2005) and also provides partial correction in some misfolding diseases (Westerheide and Morimoto, 2005). Moreover, HSF1 overexpression protects against pressure overload-induced cardiac fibrosis and dysfunction by blocking Smad3 activation (Zhou et al., 2017). In our study, the expression and deacetylation of HSF1 were decreased in the BDL group, whereas treatment with SalA increased the expression and deacetylation of HSF1 in a dose-dependent manner. Similarly, SalA significantly inversed the PDGF-BB-induced inhibition of deacetylation of HSF1 *in vitro*, which was related to the protective effect of SalA against ER stress and liver fibrosis. However, the protection of SalA was abrogated upon EX527 treatment. Accordingly, the overexpression of HSF1 markedly inhibited PDGF-BB-induced ER stress and fibrosis. All above, SalA can increase the expression and deacetylation of HSF1 both *in vivo* and *in vitro*, and attenuate liver fibrosis. Therefore, these results suggested that HSF1 plays a critical role in the pathogenesis of fibrosis and SalA enhances the expression and deacetylation of HSF1 through up-regulation of SIRT1 to protect against liver fibrosis. However, whether the protection of SalA involved other molecular mechanisms such as smad signaling pathway through SIRT1 and HSF1 is still unknown, which remains to be further explored.

In this study, we discovered that HSF1 regulates ER stress in liver fibrosis, and SalA regulates HSF1 by up-regulating SIRT1. As reported, the acetylation of HSF1 increases under heat shock conditions, thereby dampening the response, and the deacetylation of HSF1 increases the magnitude and length of the HSR (Zelin and Freeman, 2015). We found that the increased expression of SIRT1 leads to the deacetylation of HSF1 and up-regulation of heat shock proteins, which can relive liver fibrosis through inhibition of ER stress. Furthermore, the phosphorylation and ubiquitylation control of HSF1 influence its DNA binding and activities. However, the effect of these HSF1 regulation mechanisms in BDL-induced liver fibrosis remains to be further explored.

## Conclusion

Our findings indicate that ER stress promotes BDL-induced liver fibrosis, while SalA improves this progression. The protective effect of SalA on other liver fibrosis animal models (CCl4, thioacetamide, etc.) or other pro-fibrotic factors (TGF-β, etc.) induced *in vitro* model remains to be further studied. The molecular mechanism of SalA against BDL-induced liver fibrosis involves SIRT1 deacetylating HSF1, resulting in a decrease in ER stress and liver fibrosis. We demonstrated that SIRT1 activation protects against ER stress, providing grounds for its use in the development of therapies in liver fibrosis.

## Author Contributions

JY, JZ, and XT conceived and designed the study and wrote the paper. JZ, RW, TX, SZ, YZ, and ZL performed the experiments. CW, JZ, DG, and YH helped the experiments. JY provided financial support. All authors reviewed and approved the manuscript.

## Conflict of Interest Statement

The authors declare that the research was conducted in the absence of any commercial or financial relationships that could be construed as a potential conflict of interest.

## Abbreviations:

α-SMA: alpha-smooth muscle actin
ALT: alanine amino-transferase
AST: aspartate aminotransferase
ATF4: activating transcription factor 4
ATF6: activating transcription factor 6
BDL: bile duct ligation
COL1A2: collagen 1A2
ER stress: endoplasmic reticulum stress
GRP78: glucose regulated protein 78
HFD: high-fat diet
HSF1: heat shock factor 1
HSR: heat shock response
IRE-1α: inositol-requiring protein 1α
NAFLD: non-alcoholic fatty liver disease
PDGF-BB: platelet-derived growth factor-BB homodimer
PERK: protein kinase R-like ER kinase
SalA: salvianolic acid A
SIRT1: sirtuin 1
UPR: unfolded protein response.
